# Postorthodontic lower incisor and canine inclination and labial gingival recession in adult patients: A prospective study

**DOI:** 10.1007/s00056-020-00263-1

**Published:** 2020-11-25

**Authors:** Edyta Kalina, Małgorzata Zadurska, Bartłomiej Górski

**Affiliations:** 1 Department of Orthodontics, Medical University of Warsaw, Stanisława Binieckiego St 6, 02-097 Warsaw, Poland; 2 Department of Periodontology and Oral Diseases, Medical University of Warsaw, Stanisława Binieckiego St 6, 02-097 Warsaw, Poland; 3 Department of Periodontology, Medical University of Lublin, Karmelicka St 7, 20-081 Lublin, Poland

**Keywords:** Orthodontic treatment, Soft tissue, Tooth inclination, Periodontal phenotype, Tooth root, Kieferorthopädische Behandlung, Weichgewebe, Zahnneigung, Parodontaler Phänotyp, Zahnwurzel

## Abstract

**Purpose:**

The goal was to determine whether changes in the inclination of lower incisors and canines upon orthodontic treatment with fixed appliances poses a threat for labial gingival recession in adult patients.

**Methods:**

The sample of this prospective clinical trial consisted of 32 adult patients (mean age 25.08 ±6.50 years) treated with fixed appliances. Plaque and bleeding indices, probing pocket depth, clinical attachment level, gingival recession height (GR) and width (GRW), gingival thickness (GT), and keratinized tissue width were clinically recorded, while cone beam computed tomography (CBCT) was used to evaluate teeth inclination before (T1) and after treatment (T2). Oral hygiene, brushing habits, and smoking were controlled.

**Results:**

During orthodontic treatment on 15 (8.33%) teeth (10 incisors and 5 canines), spontaneous complete improvement of pre-existing GR was observed. On 2 incisors, GR decreased and on 3 teeth GR did not change. Moreover, 1 incisor presented an increased GR, while 2 teeth developed new defects. Mean GR, GRW, and GT decreased significantly only on the incisors. Proclination of incisors and canines during treatment (compared with retroclination of the teeth) implicated a lower reduction in GR at T2: 0.19 mm (*p* = 0.034) and 0.18 mm (*p* = 0.037), respectively. Multiple regression analysis confirmed that more tooth proclination was associated with a higher risk for an increase in GR (*p* < 0.00).

**Conclusion:**

Properly planned changes in lower incisor and canine inclination can be carried out in adult patients without posing a high risk to labial gingival recessions if the individual periodontal biotype is respected. The reported outcomes underscore the orthodontic principle to keep tooth roots inside the alveolar bone.

## Abstract (de)

**Zielsetzung:**

Ziel war es, festzustellen, ob Veränderungen in der Neigung der unteren Schneide- und Eckzähne nach einer kieferorthopädischen Behandlung mit festsitzenden Apparaturen eine Gefahr für eine labiale gingivale Rezession bei erwachsenen Patienten darstellen.

**Methoden:**

Die Stichprobe dieser prospektiven klinischen Studie bestand aus 32 erwachsenen, mit festsitzenden Apparaturen behandelten Patienten (Durchschnittsalter 25,08 ±6,50 Jahre). Plaque- und Blutungsindizes, Sondierungstiefe, klinischer Attachmentlevel, Höhe (GR) und Breite (GRW) der gingivalen Rezession, gingivale Dicke (GT) und Breite des keratinisierten Gewebes wurden klinisch erfasst, während die digitale Volumentomographie (DVT) zur Beurteilung der Zahnneigung vor (T1) und nach der Behandlung (T2) eingesetzt wurde. Mundhygiene, Zahnputzgewohnheiten und Rauchen wurden kontrolliert.

**Ergebnisse:**

Während der kieferorthopädischen Behandlung an 15 (8,33%) Zähnen (10 Schneide-, 5 Eckzähne) wurde eine spontane vollständige Verbesserung der bereits vorhandenen GR beobachtet. Bei 2 Schneidezähnen nahm die GR ab und bei 3 Zähnen veränderte sich die GR nicht. Darüber hinaus wies ein Frontzahn eine erhöhte GR auf, während bei 2 Zähnen neue Defekte auftraten. Die mittlere GR, GRW und GT nahmen nur bei den Schneidezähnen signifikant ab. Die Proklination der Schneidezähne und der Eckzähne während der Behandlung (im Vergleich zur Retroklination) bedeutete eine geringere Abnahme der GR bei T2: 0,19 mm (*p* = 0,034) bzw. 0,18 mm (*p* = 0,037). Eine multiple Regressionsanalyse bestätigte, dass mehr Proklination mit einem höheren Risiko für eine Erhöhung der GR verbunden war (*p* < 0,00).

**Schlussfolgerung:**

Angemessen geplante Veränderungen der unteren Schneidezahn- und Eckzahnneigung können bei erwachsenen Patienten durchgeführt werden, ohne dass ein hohes Risiko für labiale Gingivarezessionen besteht, wenn der individuelle parodontale Biotyp respektiert wird. Die berichteten Ergebnisse unterstreichen das kieferorthopädische Prinzip, die Zahnwurzeln innerhalb des Alveolarknochens zu halten.

## Introduction

The lifespan of humans is increasing, and more people are keeping more of their teeth throughout their lifetime. As a result, the rate of gingival recessions and related damage to root surfaces is increasing. Accordingly, it was reported that 50% of people aged 18–64 years and 88% of people aged 65 years or older had at least one site with recession [10]. The labial surfaces of mandibular incisors and maxillary molars were reported to be involved most often [1]. A wide range of predisposing and precipitating factors for gingival recession were suggested, among which mechanical trauma and periodontal diseases seem to be of utmost importance [7].

There is a likelihood of development or progression of gingival recessions in patients during or after orthodontic treatment [4, 13, 14, 16, 17]. The reported frequency ranges from 5–30.9% upon completion of treatment, and an increase in incidence of up to 50% in a 7-year observation period was reported. Furthermore, in a very recent systematic review, the lower incisors were found to be the most vulnerable teeth to develop labial recession [6]. Direction of tooth movement and buccolingual thickness of the gingiva were identified as significantly contributing to soft tissue response in orthodontic therapy [9, 11]. In addition, the importance of optimal buccolingual tooth inclination was underscored.

There is still an ongoing debate in the literature regarding postorthodontic lower incisor and canine inclination and its relationship to gingival recession in nongrowing patients. A recent review by Tepedino et al. [19] showed no strong evidence that orthodontic proclination of mandibular incisors increases the risk of recession development. However, as only two observational studies were included in the qualitative analysis, a meta-analysis was not feasible. Overall, the heterogeneity between studies is large, and the majority of studies are retrospective in nature, of low-to-moderate quality, and have a plethora of inadequately controlled confounders. To our knowledge, no prospective study has been published so far regarding this very important clinical issue. Thus, the focus of this study was to determine the impact of changes in lower incisor and canine inclination on labial gingival recession in adult patients treated with fixed orthodontic appliances.

## Materials and methods

This study was designed as a single-center prospective trial. All included patients were orthodontically treated with fixed appliances and underwent meticulous periodontal evaluation before and after treatment. This study was performed in accordance with the Helsinki Declaration of 1975, as revised in 2000, and it was assessed and approved by the local ethics committee (KB/236/2014).

### Study population

**Fig. 1:**
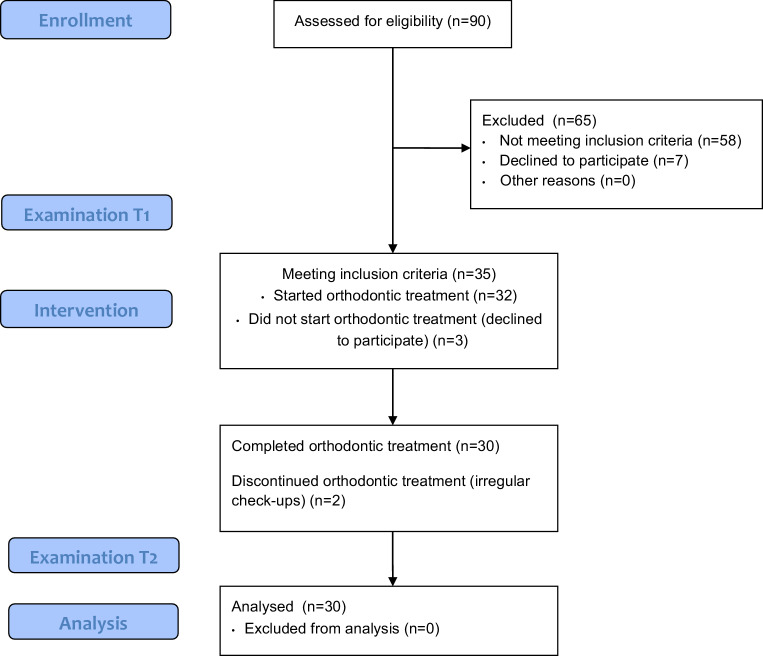
Study flow diagram (*n* number of patients, *T1* before orthodontic treatment, *T2* after orthodontic treatment) Studienablaufdiagramm (*n* Patientenzahlen, *T1* vor kieferorthopädischer Behandlung, *T2* nach kieferorthopädischer Behandlung)

Of the 90 patients who were referred to the Department of Orthodontics, Medical University of Warsaw from January 2015 to December 2015, 32 subjects met the eligibility criteria and signed informed consent for participation (Fig. 1). In all, 14 patients had skeletal class I malocclusion, 13 patients had class II malocclusion, and 5 patients had class III malocclusion (Table 1). Each participant was thoroughly informed about the study protocol. The size of the group was determined by financial limitations.

**Table 1:** Baseline sample characteristics and patients’ answers to the questionnaire Basismerkmale der Stichprobe und Antworten der Patienten auf den Fragebogen

Variables	
*Age*	25.08 (±6.50)
*Sex, n (%)*	
Male	14 (43.75)
Female	18 (56.25)
*FMPI (%)*	13.77 (±21.72)
*FMBI (%)*	14.55 (±21.83)
*Skeletal class, n (%)*	
I	14 (43.75)
II	13 (40.63)
III	5 (15.62)
*ML:NL angle, n (%)*	
High	5 (15.63)
Low	6 (18.75)
Normal	21 (65.62)
*Canine class, n (%)*	
I	18 (56.25)
II	11 (34.37)
III	3 (9.38)
*Toothbrush, n* *(%)*	
Electric	12 (37.5)
Manual	20 (62.5)
*Movements with toothbrush when brushing teeth, n* *(%)*	
Horizontal	21 (65.63)
Circular	7 (21.88)
Sweeping	4 (12.5)
*Frequency of tooth brushing, n (%)*	
≤2 × daily	19 (59.37)
>2 × daily	13 (40.63)

*n* number, *FMPI* full mouth plaque index, *FMBI* full mouth bleeding index

The inclusion criteria were as follows: age over 18 years; all mandibular incisors and canines without restorations involving the cementoenamel junction (CEJ) present in the oral cavity; no gingivitis or periodontitis; and nonsmokers. Excluded were patients with the following: previous orthodontic or surgical treatment in the anterior area of mandible; syndromes, congenital and developmental defects; maxillofacial and dental trauma cases; subjects taking medications which affect the periodontium; oral/labial piercing; and pregnant or breastfeeding women.

Following inclusion into the study, each participant completed a survey inquiring about the following: type of toothbrush used (electric or manual); movements with toothbrush when brushing teeth (horizontal, circular, sweeping); and frequency of toothbrushing (≤twice daily, >twice daily). Patients were set up for a prophylaxis appointment during which they were guided to use the roll technique in a similar manner to reduce mechanical trauma.

### Clinical examination

Patients were evaluated in two clinical examination sessions by an experienced and calibrated periodontist (B.G.): on the day of bonding prior to placing the orthodontic appliance (T1), and 3–4 weeks after a debonding and scaling session to avoid possible influence of gingivitis related to the orthodontic appliance (T2). For the calibration exercise, lower incisors and canines in 6 patients, not included in the study, were evaluated on two separate occasions, 48 h apart. Calibration was approved when ≥90% of the recordings could be reproduced within a difference of 1.0 mm, and the same measurements were repeated in 75% of the sites.

The following parameters were assessed at the lower incisors and canines (apart from plaque and bleeding indices) under local anesthesia (lignocaine hydrochloride 2%):

- Full mouth plaque index (FMPI)—percentage of tooth surfaces that exhibited plaque,
- Full mouth bleeding index (FMBI)—percentage of sites that bled from the bottom 15 s after probing at three points (mesiobuccal—MB, buccal—B, distobuccal—DB),
- Probing pocket depth (PPD)—distance from the gingival margin to the bottom of the sulcus at three points (MB, B, DB),
- Clinical attachment level (CAL)—distance from CEJ to the bottom of the sulcus at three points (MB, B, DB),
- Gingival recession height (GR)—distance from CEJ to the gingival margin mid-buccally,
- Gingival recession width (GRW)—distance measured horizontally at CEJ level,
- Gingival thickness (GT)—measured 2 mm apically to the gingival margin by perpendicular insertion of a 10 mm endodontic spreader (Poldent, Warsaw, Poland) with a silicone stopper. Each measurement was performed in triplicate to diminish inaccuracy. Sites with GT ≤ 1 were considered as thin phenotype, whereas with GT > 1 mm as thick phenotype, and
- Keratinized tissue width (KTW)—distance from the gingival margin to the mucogingival junction after staining with iodine solution.

PPD, CAL, GR, GRW, and KTW were measured using a manual periodontal probe (UNC probe 15 mm, Hu-Friedy, Chicago, IL, USA) and rounded to the nearest of 0.5 mm. An electronic caliper (YATO YT-7201, Toya, Wrocław, Poland), with 0.01 mm accuracy was selected to calculate GT.

### Evaluation of CBCTs

The cone beam computed tomographies (CBCT) were acquired on a Scanora 3Dx machine (Soredex, Nahkelantie, Tusula, Finland) with 90 kVp and 10 mA. The voxel size was 0.3 mm, while the image acquisition field was 8 × 10 cm. The assessment was carried out using OnDemand 3D™ AppProject (version 1.0.10.4304; KaVo, Biberach an der Riß, Germany) software. Radiographic analysis was performed by a calibrated clinician (E.K.). A calibration exercise was completed by examining 10 nonstudy-related CBCTs. Incisor and canine inclinations were assessed separately for each tooth before (T1) and after (T2) orthodontic treatment. The lower mandibular line (ML), defined as a line connecting points Gonion and Gnathion, was initially established on the sagittal cross section of the mandible set at 100 mm. Then, a curve crossing the maximum labial and lingual convexity at the level of the CEJ was drawn and an axial slice was obtained. Subsequently, a line was drawn along the long axis of the tooth in the middle of the root canal on the coronal image. The measurements of angles between the incisors’ and canines’ long axes and ML were made on the sagittal image of the evaluated teeth: 33, 32, 31, 41, 42, and 43. All calculations were carried out twice, 48 h apart.

### Orthodontic treatment

All patients were diagnosed and treated by one orthodontist (E.K). On the basis of cephalomertic radiographs, skeletal class (ANB) and ML:NL angle were determined. Interproximal enamel reduction or tooth extractions were planned in patients with severe crowding, incisor protrusion, and thin phenotype to preserve the teeth within the dentoalveolar envelope and to avoid overexpansion of the arch. Straight wire technique with the use of a 0.022’’ (inch) slot, Roth prescribed brackets (Avex Mx, Opal Orthodontics, South Jordan, UT, USA) and nickel–titanium (NiTi) and stainless steel (SS) wires (Tru Force, Ortho Technology, Lutz, FL, USA) were applied. Treatment was meticulously designed for each clinical scenario. Patients’ optimal oral hygiene was ensured during control visits and care was taken to keep FMPI and FMBI as low as possible. If required, patients were provided with scaling and polishing.

### Statistical analysis

All statistical analyses were carried out using Statistica 13 (StatSoft Polska, Cracow, Poland). Statistical descriptions, consisting of mean and standard deviation (SD), were calculated for the measured parameters. Normality of data distribution was evaluated and confirmed by visual inspection of histograms, owing to the small sample size. To assess the agreement and precision between two measurements of inclination (intrarater reliability), the Bland–Altman plot was used. T‑test for dependent means was used for intragroup (changes within incisors or canines over time) and intergroup (differences between incisors and canines at the same time point) comparison. In addition, the post hoc power of the study when applying T‑tests was calculated. Analysis of variance (ANOVA) was performed to check the impact of orthodontic tooth movement and final tooth position on clinical periodontal parameters. Spearman correlation coefficients (R) were calculated to determine correlations between orthodontic movements and clinical periodontal parameters for the lower incisors and canines. The influence of tooth proclination, age, and sex on changes in GR was examined utilizing multiple regression analysis. Odds ratio (OR) was used to assess the risk of developing labial recession after a definite proclination (T2–T1). The threshold for statistical significance was set at *p* < 0.05.

## Results

**Fig. 2:**
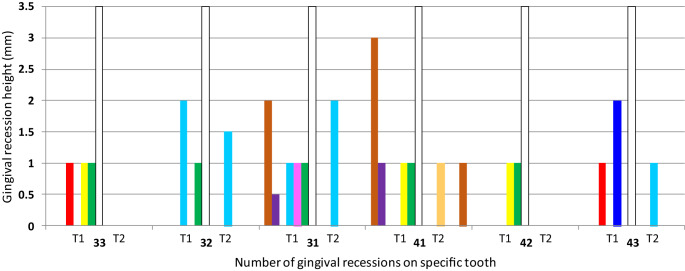
Changes in gingival recession height during the course of treatment (*T1* before orthodontic treatment, *T2* after orthodontic treatment). Recessions in individual patients are represented by different colors: I–*green*, II–*yellow*, III–*light blue*, IV–*red*, V–*purple*, VI–*brown*, VII–*pink*, VIII–*orange*, IX–*dark blue* and are aligned with patients described in Table 5 Veränderungen der Höhe der gingivalen Rezession im Verlauf der Behandlung (*T1* vor der kieferorthopädischen Behandlung, *T2* nach der kieferorthopädischen Behandlung). Rezessionen bei einzelnen Patienten werden durch unterschiedliche Farben dargestellt: I‑*grün*, II-*gelb*, III-*hellblau*, IV-*rot*, V‑*violett*, VI-*braun*, VII-*pink*, VIII-*orange*, IX-*dunkelblau* und sind auf die in Tab. 5 beschriebenen Patienten ausgerichtet

At T1, the study group involved 32 patients, 18 women (56.26%) and 14 men (43.76%), aged 25.08 (±6.50) years. Due to irregular appointments during orthodontic treatment, 2 patients were excluded from the final evaluation. Overall, 180 mandibular teeth, including 120 incisors and 60 canines in 30 patients (18 women, 60%; 12 men, 40%; aged 26.05 ±5.01 years) were analyzed at T2. Characteristics of the sample at baseline are presented in Table 1. Gingival recessions were detected in 8 (26.67%) patients, 15 (12.5%) on incisors and 6 (10%) on canines in the baseline evaluation (Fig. 2). Treatment lasted an average of 23.1 (±4.57) months. In all, 11 patients (34.3%) were treated with premolar extractions. At T2, FMPI was 14.52 (±20.99) % and FMBI was 15.75 (±21.16) %. During orthodontic treatment on 15 (8.33%) teeth, 10 incisors and 5 canines, spontaneous complete recession coverage was observed. Whereas on 2 (1.66%) incisors GR diminished and on 3 (1.66%) teeth (2 incisors and 1 canine) GR did not change. The changes in gingival recession height during the course of treatment are shown in Fig. 2. GR increased on only 1 (0.55%) incisor, while new defects developed on 2 (1.11%) teeth (one incisor and one canine). Overall, at T2 only 4 (13.33%) patients and 8 teeth (4.44%) presented with GR.

**Fig. 3:**
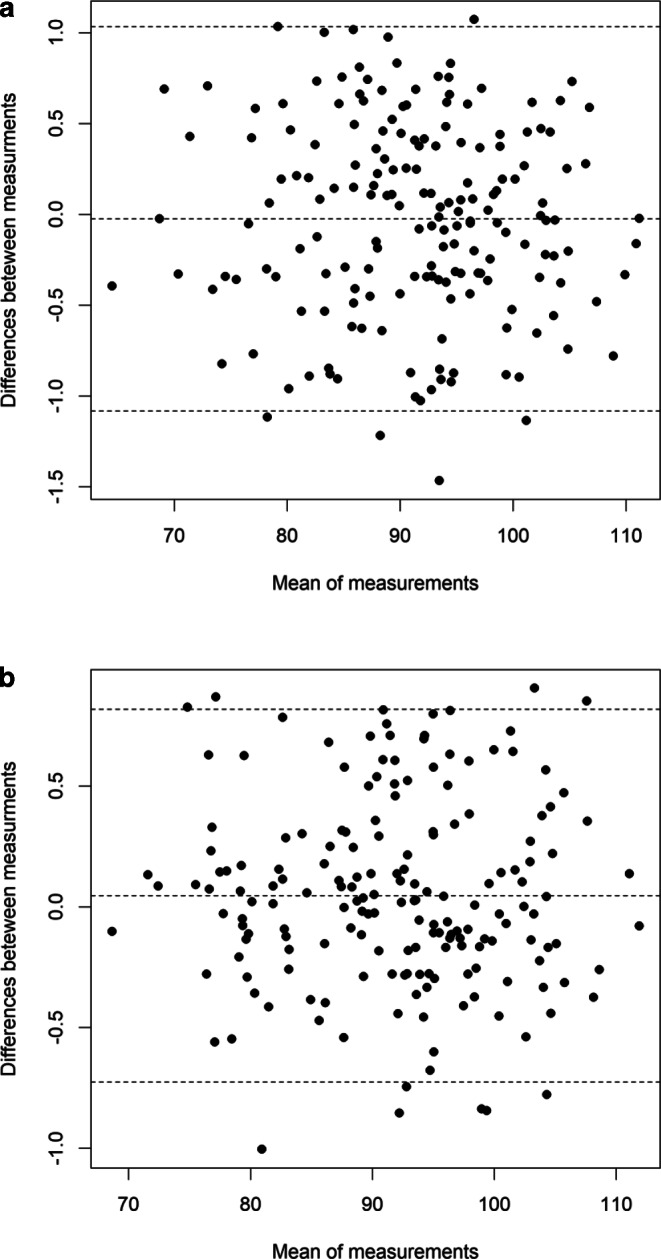
Bland–Altmann plot for the measurements of inclination performed **a** before and **b** after orthodontic treatment Bland-Altmann-Diagramm für die Inklinationsmessungen, **a** vor und **b** nach der kieferorthopädischen Behandlung

Table 2 depicts baseline and postorthodontic treatment data for the clinical periodontal parameters. With treatment, GR, GRW, and GT decreased significantly for the incisors. A significant increase in PPD and a gain in CAL mid-buccally was observed for incisors and canines. Despite the fact that these results were statistically significant, the statistical post hoc power analysis for this part of the study revealed an average level. The initial and final mean value of inclination was significantly larger for the incisors than for the canines (Table 3). The level of agreement for the measurements (intraexaminer reliability) was good and no systematic errors were observed (Fig. 3).

**Table 2:** Changes in clinical periodontal parameters Änderungen bei klinischen Parodontalparametern

Variables	Before treatment (T1)	After treatment (T2)	∆ T2–T1	*p*-value
*GR*				
32–42	0.16 ± 0.48	0.07 ± 0.32	−0.09 ± 0.38	0.010* (23.45%)
33, 43	0.13 ± 0.43	0.05 ± 0.28	−0.08 ± 0.38	0.096 (10.21%)
*P*	0.719 (3.45%)	0.690 (3.11%)	0.891 (2.21%)	–
*GRW*				
32–42	0.23 ± 0.68	0.09 ± 0.45	−0.14 ± 0.59	0.010* (24.21%)
33, 43	0.31 ± 0.94	0.08 ± 0.46	−0.22 ± 0.92	0.061 (13.89%)
*P*	0.539 (4.21%)	0.879 (2.34%)	0.463 (5.21%)	–
*GT*				
32–42	1.03 ± 0.42	0.96 ± 0.42	−0.06 ± 0.33	0.042* (15.23%)
33, 43	0.96 ± 0.37	0.91 ± 0.38	−0.04 ± 0.24	0.157 (8.93%)
*P*	0.280 (7.81%)	0.427 (5.30%)	0.702 (3.33%)	–
*KTW*				
32–42	3.89 ± 1.37	3.83 ± 1.63	−0.05 ± 1.01	0.530 (4.89%)
33, 43	2.77 ± 1.01	2.84 ± 1.31	0.07 ± 0.98	0.555 (4.92%)
*P*	0.000* (95.61%)	0.000* (73.71%)	0.401 (5.34%)	–
*PPD B*				
32–42	1.27 ± 0.l4	1.54 ± 0.59	0.27 ± 0.72	0.000* (68.41%)
33, 43	1.47 ± 0.56	1.49 ± 0.59	0.01 ± 0.73	0.859 (2.35%)
*P*	0.011* (22.21%)	0.593 (4.65%)	0.041 (14.89%)	–
*PPD*				
32–42	1.97 ± 0.46	2.27 ± 0.53	0.32 ± 0.52	0.000* (64.95%)
33, 43	2.08 ± 0.55	2.30 ± 0.55	0.22 ± 0.59	0.005* (34.23%)
*P*	0.547 (4.72%)	0.661 (4.21%)	0.876 (2.14%)	–
*CAL B*				
32–42	0.63 ± 0.94	0.37 ± 0.78	−0.26 ± 0.87	0.001* (21.43%)
33, 43	0.51 ± 0.94	0.30 ± 0.77	−0.21 ± 0.69	0.023* (15.54%)
*P*	0.397 (6.55%)	0.558 (4.39%)	0.637 (4.05%)	–
*CAL*				
32–42	0.44 ± 0.67	0.37 ± 0.51	−0.06 ± 0.63	0.261 (7.32%)
33, 43	0.35 ± 0.63	0.36 ± 0.48	0.00 ± 0.50	0.897 (2.40%)
*P*	0.402 (5.45%)	0.281 (7.52%)	0.692 (4.32%)	–

A negative value for ∆ T2–T1 indicates a reduction in GR, GRW, GT, KTW, and a gain in CAL

T‑test for dependent means was used for intragroup and intergroup comparison. In brackets the calculated post hoc power of test is reported

*T1* before orthodontic treatment, *T2* after orthodontic treatment, *GR* gingival recession height, *GRW* gingival recession width, *GT* gingival thickness, *KTW* keratinized tissue width, *PPD B* probing pocket depth mid-buccally, *PPD* probing pocket depth at three evaluated points, *CAL B* clinical attachment level mid-buccally, *CAL* clinical attachment level at three evaluated points

*Statistically significant (*p* ≤ 0.05)

**Table 3:** Changes in labiolingual inclination Veränderungen bei der labiolingualen Neigung

Tooth type	Before treatment (T1)	After treatment (T2)	∆ T2–T1	*p*-value
32:ML–42:ML	93.26 ± 8.84	94.01 ± 9.12	0.74 ± 8.12	0.317
33:ML, 43:ML	87.72 ± 8.46	87.79 ± 7.13	0.07 ± 8.38	0.948
*P*	0.000*	0.000*	0.606	–

*T1* before orthodontic treatment; *T2* after orthodontic treatment, *32:ML* angle between long axis of tooth 32 and mandibular plane (Gonion-Gnathion), *42:ML* angle between long axis of tooth 42 and mandibular plane, *33:ML* angle between long axis of tooth 33 and mandibular plane, *43:ML* angle between long axis of tooth 43 and mandibular plane

*Statistically significant (*p* ≤ 0.05)

Changes in the clinical periodontal parameters related to the orthodontic movements are presented in Table 4. Overall, 66 incisors and 24 canines were proclined, 43 incisors and 27 canines underwent retroclination, and 11 incisors and 9 canines did not change their inclination. Significantly greater reductions in GR and GRW and a larger CAL gain mid-buccally were observed in cases where incisors and canines were retroclined, when compared with proclined teeth. Changes in gingival recession dimensions and labiolingual tooth inclinations are listed in Table 5. Individual patients where changes in gingival recession occurred were selected. Teeth that showed improvement in gingival recession had a mean change in inclination of −7.20 ±8.99°, whereas teeth in which recession increased had a mean change in inclination of 7.60 ±9.29° (Table 6).

**Table 4:** Impact of orthodontic movement on changes in clinical periodontal parameters Einfluss der kieferorthopädischen Bewegung auf Veränderungen der klinischen parodontalen Parameter

Tooth type	*n* (%)	Changes in periodontal parameters (T2–T1)					
		∆ GR	∆ GRW	∆ GT	∆ KTW	∆ PPD B	∆ CAL B
*32–42*							
Proclination ∆ > 1°	66 (55.0)	−0.01 ± 0.19	−0.01 ± 0.26	−0.03 ± 0.28	−0.12 ± 0.91	0.21 ± 0.65	−0.01 ± 0.65
Retroclination ∆ < −1°	43 (35.8)	−0.20 ± 0.55	−0.32 ± 0.86	−0.07 ± 0.42	0.02 ± 1.17	0.41 ± 0.79	−0.61 ± 1.02
Alignment ∆ = [−1°, 1°]	11 (9.2)	−0.09 ± 0.30	−0.18 ± 0.60	−0.21 ± 0.21	0.04 ± 0.96	0.09 ± 0.83	−0.36 ± 1.02
*P*-value	–	0.034*	0.027*	0.262	0.704	0.236	0.002*
R (*p*-value)	–	0.234 (0.010*)	0.201 (0.028*)	−0.014 (0.882)	−0.143 (0.119)	−0.048 (0.601)	0.299 (0.001*)
*33, 43*							
Proclination ∆ > 1°	24 (40.0)	0.04 ± 0.20	0.08 ± 0.40	−0.02 ± 0.20	0.20 ± 1.25	0.04 ± 0.80	0.04 ± 0.60
Retroclination ∆ < 1°	27 (45.0)	−0.22 ± 0.50	−0.57 ± 1.23	−0.03 ± 0.26	−0.03 ± 0.77	−0.11 ± 0.64	−0.42 ± 0.71
Alignment ∆ = [−1°, 1°]	9 (15.0)	0.00 ± 0.00	0.00 ± 0.00	−0.14 ± 0.30	0.06 ± 0.77	0.37 ± 0.74	−0.25 ± 0.70
*P*-value	–	0.037*	0.027*	0.501	0.682	0.253	0.053
R (*p*-value)	–	0.417 (0.001*)	0.419 (0.001*)	−0.091 (0.493)	−0.051 (0.702)	0.049 (0.711)	0.295 (0.023*)

A negative value for ∆ T2–T1 indicates a reduction in GR, GRW, GT, KTW, an increase in PPD and a gain in CAL

ANOVA test was used for analysis of differences between changes in clinical periodontal parameters with regard to tooth inclination after orthodontic treatment

Spearman’s correlation coefficients (R) were calculated to determine correlations between tooth inclination and changes in clinical periodontal parameters

*T1* before orthodontic treatment, *T2* after orthodontic treatment, *n* number of teeth, *GR* gingival recession height, *GRW* gingival recession width, *GT* gingival thickness, *KTW* keratinized tissue width, *PPD B* probing pocket depth mid-buccally, *CAL B* clinical attachment level mid-buccally

*Statistically significant (*p* ≤ 0.05)

**Table 5:** Changes in gingival recession and labiolingual tooth inclination for individual patients Veränderungen der gingivalen Rezession und der labiolingualen Zahninklination bei einzelnen Patienten

Patient	Tooth	GR T1	GR T2	∆ GR T2–T1	Inclination T1	Inclination T2	∆ Inclination T2–T1
I	33	1	0	−1	83.2	80.8	−2.4
	32	1	0	−1	96.5	81.6	−14.9
	31	1	0	−1	93.2	81.8	−11.4
	41	1	0	−1	96.6	79.4	−17.2
	42	1	0	−1	97.2	79.8	−17.4
II	33	1	0	−1	95.9	72.5	−23.4
	41	1	0	−1	83.7	76.8	−6.9
	42	1	0	−1	81.5	80.9	−0.6
III	32	2	1.5	−0.5	98.9	103.9	5
	31	1	2	1	95.6	104.6	9
	43	0	1	1	76.6	91.7	15.1
IV	33	1	0	−1	97.6	83.1	−14.5
	43	1	0	−1	94.1	87.4	−6.7
V	31	0.5	0	−0.5	94.4	100.7	6.3
	41	1	0	−1	94.6	103.2	8.6
VI	31	2	0	−2	96.1	84.6	−11.5
	41	3	1	−2	96.1	88.5	−7.6
VII	31	1	0	−1	94.64	92.9	−1.74
VIII	41	0	1	1	78.1	76.8	−1.3
IX	43	2	0	−2	102.5	96.5	−6

A negative value for ∆ GR indicates a reduction in GR; a negative value for ∆ Inclination indicates tooth retroclination

*T1* before orthodontic treatment, *T2* after orthodontic treatment, *GR* gingival recession height, *Inclination* angle between long axis of tooth and mandibular plane (Gonion-Gnathion)

**Table 6:** Mean changes in tooth inclination based on changes in gingival recession height Mittlere Änderungen der Zahninklination basierend auf Änderungen der Höhe der gingivalen Rezession

GR changes (number of teeth)	Inclination T1	Inclination T2	∆ Inclination T2–T1
GR T1 > GR T2 (17)	93.93 ± 5.73	86.73 ± 9.48	−7.20 ± 8.99
GR T1 < GR T2 (4)	83.43 ± 10.56	91.03 ± 13.91	7.60 ± 8.29
GR T1 = GR T2 (159)	94.33 ± 10.91	92.67 ± 11.90	−1.67 ± 8.56

A negative value for ∆ Inclination indicates tooth retroclination

*T1* before orthodontic treatment, *T2* after orthodontic treatment, *GR* gingival recession height, *Inclination* angle between long axis of tooth (33–43) and mandibular plane (Gonion-Gnathion)

Multiple regression analysis evaluating the impact of age, sex, and increase in lower incisor and canine proclination (T2–T1) on the change in gingival recession height confirmed that a greater proclination of teeth bore a higher risk for an increase in recession. However, neither age nor sex influenced posttreatment soft tissue margin position (Table 7).

**Table 7:** Multiple regression analysis evaluating the significance of age, sex, and increase in lower incisor and canine proclination on changes in labial gingival recession (T2–T1) Multiple Regressionsanalyse zur Bewertung der Bedeutung von Alter, Geschlecht und Zunahme der Proklination der unteren Schneidezähne und der Eckzähne bei Veränderungen der labialen gingivalen Rezession (T2–T1)

Independent variables	Coefficient b	Standard error	*p*-value
Age	−0.002	0.004	0.623
Sex	0.113	0.063	0.038
Tooth proclination T2–T1 (°)	0.018	0.003	<0.000*

Significance of the model: R = 0.398, R^2^ = 0.158, *p* < 0.00001. Sex: 0 = male, 1 = female. Dependent variable (Y): Change in labial gingival recession height T2–T1. Multiple regression analysis: Y = −0.114711 + b_1_age + b_2_sex + b_3_tooth proclination (T2–T1)

*T1* before orthodontic treatment, *T2* after orthodontic treatment

## Discussion

Due to the steadily increasing demand for orthodontic treatment in adults, the potential impact of orthodontic tooth movement on gingival recessions represents a substantial clinical problem. In our study, only few gingival recessions were observed, and most of them affected the central incisors. Changing the axial inclination of these teeth influenced the amount of gingival recession. Thus, for proclined incisors and canines, when compared with retroclined teeth, the reduction in GR was 0.19 and 0.18 mm lower, respectively. However, tooth proclination did not play a major role, as the above mentioned values were small and thus of vague clinical relevance.

At the same time, a significant decrease in GT could also be expected, while KTW did not change noticeably. This observation may be explained, at least partially, by the adopted periodontal evaluation. GT was measured 2 mm apically to the gingival margin, and when the tooth was retroclined, its root moved in the labial direction (thus, the value of GT might be lower). Another finding that could seem contradictory is an increase in PPD accompanied by a gain in CAL. It may be associated with occasionally observed slight and reversible gingival enlargement during orthodontic treatment. An attempt was made to eliminate this effect by performing the clinical examination 3–4 weeks after the debonding procedure. Nevertheless, the risk of gingival recession development or increase following the reversion of the gingival enlargement after this time should be considered. Teeth that showed improvement in terms of gingival recession showed a mean change in inclination of −7.20°, while teeth in which GR increased showed a mean change in inclination of 7.60°. Although changes in GR, GRW, GT at T1 and T2 were statistically significant, the statistical post hoc power of the study appeared to be low. The possible reason for this was the small number of teeth with gingival recession and high variability of GT values. Although the study group involved 30 patients, it has to be underlined that observations were made separately on 180 teeth. A similar number of patients were included in another prospective study [15]. The present data implicate that meticulously planned orthodontic treatment may lead to labial gingival recession improvement and a gain in CAL on lower incisors and canines.

The biological foundations of gingival recession formation during orthodontic therapy are not entirely clear. The risk of recession has traditionally been associated with preceding alveolar bone dehiscence [21]. As observed in animal studies, orthodontic tooth movement out of the alveolar bone housing resulted in the loss of marginal bone and connective tissue attachment [5, 18]. It could be speculated that due to tooth proclination, its root approaches the cortical bone, and hence leads to bone thinning or dehiscence development, which in turn might lead to gingival recession [8]. Despite that, controlled proclination is a noteworthy alternative to extraction when considering different therapeutic options in adult patients.

Even though a vast array of studies have reported the effect of mandibular incisor proclination on gingival recession, findings have been conflicting [4, 9]. Some of the above-mentioned research supports our observations, but only one study was prospective in nature [15]. Rasperini et al. [15] analyzed 60 mandibular incisors in 16 patients who had undergone orthodontic treatment. Subjects who presented with a thin periodontal phenotype were more susceptible to gingival recession, regardless of the type of orthodontic movement. Both thin periodontal phenotype and incisor proclination led to a loss of KTW (−0.50 ± 0.71 mm, *p* = 0.03; −0.67 ± 0.30 mm, *p* = 0.003; respectively). In fact, only two studies focused entirely on nongrowing subjects. Allais and Melsen [2] demonstrated that mandibular incisors in adult patients after orthodontic treatment showed more GR than in untreated controls (35 and 17%, respectively; *p* < 0.05), but it was only observed for the right central incisor and the left lateral incisor. New recessions developed in 10% of the proclined teeth, but improved in 5%. In a second study, 24 adult patients were treated with fixed appliances and extractions of first lower premolars, whereby 26 subjects were treated without extractions [20]. After treatment, there was an increase in clinical crown length of 0.37 mm for incisors and 0.84 mm for canines. The correlation between incisor inclination and their crown length was statistically significant (*p* = 0.027), which agrees with the results from the present study. Antonarakis et al. [3] followed 55 patients over an average of 4.5 years after orthodontic treatment, reporting an increase in the labial gingival recessions. Multiple regression analysis showed that younger individuals, girls, and patients with greater incisor proclination were at higher risk of recession. Moreover, incisors proclined ≥10° showed a roughly 2‑fold increase in the risk of presenting labial gingival recession (OR 2.4; 95% confidence interval = 1.1–5.4). Quite similarly, multiple regression analysis carried out in our study confirmed that more proclined teeth had a higher risk for increased in recession following orthodontic treatment, but neither age nor sex influenced the soft tissue margin position. Gingival recession decreased in case of the tooth being retroclined by roughly −7.20°, and increased when the tooth was proclined by about 7.60°. Unfortunately, the low prevalence of gingival recession in our sample precluded an evaluation of relative risk (OR). Similar methodology was adopted in previous research [14]. Before treatment, authors observed at least one labial gingival recession in 11.5% of patients older than 20 years at baseline. This value increased to 30.8% after orthodontic treatment and central incisors showed more recessions than lateral incisors. Excessive mandibular incisor proclination (≥10°) was associated with the development of recessions in 25% of the subjects, but only left central incisor demonstrated a higher risk of buccal recession (OR 1.12; 95% confidence interval = 1.01–1.23; *p* = 0.03).

On the other hand, some authors observed no association between mandibular incisor proclination and an increase in labial gingival recession. All systematic reviews on this topic have concluded that robust evidence is lacking [4, 9, 19]. Thus, our study contributes to this body of knowledge. Melsen and Allais [12] evaluated the prevalence and severity of recession of labially moved mandibular incisors in adult patients. The frequency of recession greater than 0.1 mm increased from 21% at baseline to 35% after treatment (*p* < 0.05). GR exceeding 2 mm was found in 2.8% of patients, but the increase in mean GR was not significant. It appeared that the alveolar envelope could bear incisor proclination up to a certain point before attachment loss occurred. Renkema et al. [15] followed 117 patients with bonded retainers placed immediately after orthodontic therapy for a period of 5 years. The mean increase in clinical crown height of lower incisors varied from 0.75 to 0.83 mm in the nonproclined and proclined groups, respectively (*p* = 0.273). In another study in adolescents, no relationship between lower incisor proclination during therapy and posttreatment gingival recession was observed [13]. However, the possible impact of vertical growth of alveolar process on the outcomes could not be ruled out, as the ability of the periodontal tissues to withstand orthodontic forces appears to diminish with age [16]. These contradictory findings might be explained by methodological issues such as different sample composition or the moment of evaluation. It should also be stressed that retrospective studies do not provide high-level evidence of causal relationships.

Possible limitations that should be taken into account when interpreting the reported data. Lack of randomization may be considered the main drawback of this study. The sample size might be judged as small, so the results should be interpreted with caution. Moreover, subjects were not matched by occlusal characteristics and the decision of tooth extraction depended on analysis of individual cases. However, to purposely procline or extract teeth in some patients would be ethically questionable. Second, there was no control group. Third, it may be questioned whether the sagittal canine inclination is an adequate measure. Canines are located at the corner of the mouth, and thus it may be relevant to evaluate the inclination of these teeth in the transversal plane. Fourth, the etiology of gingival recession is multifactorial. In this study, some of the confounders such as the patient’s oral hygiene, brushing habits, and smoking were controlled; thus, the risk of potential bias was diminished. It should be highlighted that meticulous clinical examination of periodontal tissues was carried out, and three-dimensional radiographs were used to evaluate each tooth movement separately during orthodontic therapy. Another important limitation is the follow-up period, as gingival recessions might occur several years after debonding. Be that as it may, a long-term follow-up of the investigated population is intended. Therefore, further well-controlled prospective studies on a larger sample and with a longer observation period are needed to elucidate the presented outcomes and identify other predictors of labial recession. The definable value of incisor and canine proclination inducing gingival recession should be determined in the future.

## Conclusion

Properly planned changes in lower incisor and canine inclination can be carried out in adult patients without posing a high risk to labial gingival recessions if the individual periodontal biotype is respected. The reported outcomes underscore the orthodontic principle to keep tooth roots inside the alveolar bone.
